# Effects of oral phosphatidic acid feeding with or without whey protein on muscle protein synthesis and anabolic signaling in rodent skeletal muscle

**DOI:** 10.1186/s12970-015-0094-7

**Published:** 2015-08-16

**Authors:** C. Brooks Mobley, Troy A. Hornberger, Carlton D. Fox, James C. Healy, Brian S. Ferguson, Ryan P. Lowery, Rachel M. McNally, Christopher M. Lockwood, Jeffrey R. Stout, Andreas N. Kavazis, Jacob M. Wilson, Michael D. Roberts

**Affiliations:** School of Kinesiology, Auburn University, Auburn, AL USA; Department of Comparative Biosciences, University of Wisconsin-Madison, Madison, WI USA; Department of Health Sciences and Human Performance, University of Tampa, Tampa, FL USA; 4LIFE Research, LLC, Sandy, UT USA; Human Performance Laboratory, University of Central Florida, Orlando, FL USA

## Abstract

**Background:**

Phosphatidic acid (PA) is a diacyl-glycerophospholipid that acts as a signaling molecule in numerous cellular processes. Recently, PA has been proposed to stimulate skeletal muscle protein accretion, but mechanistic studies are lacking. Furthermore, it is unknown whether co-ingesting PA with other leucine-containing ingredients can enhance intramuscular anabolic signaling mechanisms. Thus, the purpose of this study was to examine if oral PA feeding acutely increases anabolic signaling markers and muscle protein synthesis (MPS) in gastrocnemius with and without whey protein concentrate (WPC).

**Methods:**

Overnight fasted male Wistar rats (~250 g) were randomly assigned to four groups: control (CON, n = 6-13), PA (29 mg; *n* = 8), WPC (197 mg; *n* = 8), or PA + WPC (*n* = 8). Three hours post-feeding, gastrocnemius muscle was removed for markers of Akt-mTOR signaling, gene expression patterns related to skeletal muscle mass regulation and metabolism, and MPS analysis via the SUnSET method.

**Results:**

Compared to CON rats, PA, WPC and PA + WPC resulted in a significant elevation in the phosphorylation of mTOR (Ser2481) and rps6 (Ser235/236) (*p* < 0.05) in the gastrocnemius though there were no differences between the supplemented groups. MPS levels in the gastrocnemius were significantly (*p* < 0.05) elevated in WPC versus CON rats, and tended to be elevated in PA versus CON rats (*p* = 0.08), though MPS was less in PA + WPC versus WPC rats (*p* < 0.05) in spite of robust increases in mTOR pathway activity markers in the former group. C_2_C_12_ myoblast data agreed with the *in vivo* data herein showing that PA increased MPS levels 51 % (*p* < 0.001) phosphorylated p70s6k (Thr389) levels 67 % (*p* < 0.001).

**Conclusions:**

Our results are the first *in vivo* evidence to demonstrate that PA tends to increases MPS 3 h post-feeding, though PA may delay WPC-mediated MPS kinetics within a 3 h post-feeding window.

## Background

Skeletal muscle growth is controlled by several intricate processes dictated in large part by nutrient and intramuscular mechano-responsive factors [1]. To this end, the mammalian target of rapamycin (mTOR) is thought to be a nodal point for controlling skeletal muscle hypertrophy. While mTOR activation is complex, a simplistic overview of this process is as follows [2]: a) mTOR complex 1 (mTORC1), which is comprised of mTOR-Raptor- mLST8, can be activated by muscle contraction and nutritional factors; b) activated mTORC1 then acts to phosphorylate and activate p70s6 kinase (p70s6k) while hyper-phosphorylating eukaryotic initiation factor 4E (eIF4E)-binding proteins (4EBP-1/2); c) activated p70s6k phosphorylates and activates ribosomal protein s6 (rps6), while hyperphosphorylated 4EBP1/2 become inactive thus facilitating ribosomal assembly; and d) activated rps6 further increases ribosomal assembly via enhanced 5’-cap-dependent messenger RNA (mRNA) translation of selective genes. These aforementioned processes ultimately result in an elevation in skeletal muscle protein synthesis (MPS) and, if stimulated repetitively with resistance exercise and ample nutrition, lead to an increase in skeletal muscle accretion.

Phosphatidic acid (PA) is a diacyl-glycerophospholipid that is enriched in eukaryotic cell membranes and it can act as a signaling lipid [3]. For instance, it has been shown that PA regulates a wide array of cellular processes including but not limited to proliferation, differentiation, survival signaling, cytoskeletal rearrangement and vesicular trafficking (reviewed in [4]). PA can also elicit anabolic responses in cells. In this regard, a hallmark mechanistic study performed by Fang et al. [5] demonstrated that PA activates mTOR by binding to the FKBP12-rapamycin binding (FRB) domain on mTOR. Follow-up studies by Hornberger et al. [6] revealed that mechanical stretching of skeletal muscle promotes an increase in intramuscular PA levels and this effect was associated with the activation of mTOR signaling (e.g. increased p70s6k phosphorylation), and that eccentric contraction increases skeletal muscle PA levels which, in turn, activate mTOR signaling [7]. More recent data from Hornberger’s group suggests that the ζ isoform of diacylglycerol kinase, which synthesizes intramuscular PA via the phosphorylation of diacylglycerol, is largely responsible for the increase in PA levels and the activation of mTOR signaling that occurs in response to stretch [8]. However, others have suggested that PA does not directly bind to mTOR, but rather activates the MAP kinase pathway proteins Erk1/2 via its conversion into lysophosphatidic acid (LPA) which ultimately results in mTOR activation [9]. Likewise, others have shown that PA binds to p70s6k to exhibit its biological effects in an mTOR-independent fashion [10]. Notwithstanding, and in spite of these divergent findings, there is ample *in vitro* evidence to suggest that PA increases mTORC1 signaling.

Given the ability of biosynthesized PA to activate the intramuscular mTORC1 signaling, there is intense interest for the potential of PA supplementation to act as an ergogenic/muscle-building aid. To this end, two recent human studies supplemented participants with 750 mg of soy-derived PA over an 8-week resistance training period. Hoffman et al. [11] reported that PA supplementation increased whole-body lean body mass (LBM) by 1.7 kg, whereas the placebo group demonstrated no relative change in LBM (0.1 kg; *p* = 0.065 between groups). Joy et al. [12] performed a similar 8-week study with more participants and supervised training sessions, and reported that soy-derived PA supplementation significantly increased LBM by 2.4 kg, whereas the placebo group demonstrated marginal increases in LBM (1.2 kg; *p* < 0.05 between groups). Joy et al. also used an *in vitro* approach to demonstrate that stimulating differentiated myoblasts with soy-derived PA elicited over a 6-fold increase in p70s6k (Thr389) phosphorylation which is indicative of mTOR activation. While both studies suggest that soy-derived PA supplementation is effective at augmenting resistance exercise-induced skeletal muscle hypertrophy, neither study measured the effects of PA on MPS levels and/or mTOR signaling events *in vivo*. Likewise, it remains to be determined if other mTOR-modulating ingredients (i.e., intact dietary protein sources rich in L-leucine) provide a synergistic effect with regards to activating mTOR and downstream events related to increasing MPS. Leucine activates mTOR through RAG GTPases which, in the presence of leucine, bind to Raptor and increase mTORC1 complex localization to vesicles to increase its kinase activity [13]. PA is thought to independently activate mTOR through competitive binding with the mTOR inhibitor FKBP38; both which bind to the FRB domain [14]. Thus, given that these are two modes whereby mTORC1 kinase activity can be independently activated, it stands to reason that whey protein could synergistically activate mTOR if co-ingested with PA.

Therefore, the purpose of this study was to examine if PA acutely increases anabolic signaling markers and muscle protein synthesis (MPS) in gastrocnemius with and without whey protein concentrate (WPC) supplementation. As a side, we also sought to examine the skeletal muscle mRNA response to PA and/or WPC ingestion in an exploratory manner given that no information to our knowledge exists on how PA ingestion affects the skeletal muscle transcriptomic response. To this end, and independent of mTOR signaling and MPS, we examined key genes involved in muscle mass maintenance [myostatin (Mstn) and p21Cip1], metabolism (PGC-1α and GLUT-4), and skeletal muscle atrophy (Atrogin-1 and MuRF-1) in an exploratory manner to examine if PA with or without whey protein affected these markers.

## Methods

### Animals and feeding protocols

All experimental procedures described herein were approved by Auburn University’s Institutional Animal Care and Use Committee (protocol# 2013–2378). Male Wistar rats (~250 g) were purchased from Harlan Laboratories and were allowed to acclimate in the animal quarters for 5 days prior to experimentation. Briefly, animal quarters were maintained on a 12 h light: 12 h dark cycle, at ambient room temperature, and water and standard rodent chow (18.6 % protein, 44.2 % carbohydrate, 6.2 % fat; Teklad Global #2018 Diet, Harlan Laboratories, Indianapolis, IN, USA) was provided to animals *ad libitum*.

The day prior to the acute feeding experiments, food was removed from home cages resulting in an 18 h overnight fast. The morning of experimentation, animals were removed from their quarters between 0800–0900, transported to the Molecular and Applied Sciences Laboratory and were allowed to acclimate for approximately 3–5 h. Thereafter, rats were gavage-fed either:

1. CON: 1 ml of tap water
2. PA: 0.029 g soy-derived PA (S-PA, Mediator®, Chemi Nutra, Austin, TX, USA) suspended in 1 ml of tap water; this being a human equivalent dose of 1.5 g per the species conversion calculations of Reagan-Shaw et al. [15]
3. WPC: 0.193 g WPC (standardized to 80 %, donated graciously by C.M.L.) suspended in 1 ml of tap water; this being a human equivalent dose of 10 g
4. PA + WPC: 0.029 g soy-derived PA + 0.193 g WPC suspended in 1 ml of tap water

Of note, select data from group #3 above (WPC) published as control group in a previously published study by our group [16], but was used as a reference to examine how PA and PA + WPC affected post-feeding markers of muscle anabolism relative to WPC alone. It should also be noted that, while a 10 g human equivalent dose may be considered too low with regards to being able to increase skeletal muscle MPS given that 10 g of WPC delivers ~1 g of leucine to humans, we have consistently observed this dose of whey protein to increase MPS in rat skeletal muscle in pilot experiments (Additional file 1: Figure S1 in Mobley et al. [16]) as well as previously published data [16].

The gavage feeding procedure involved placing the animals under light isoflurane anesthesia for approximately 1 min while gavage feeding occurred. Following gavage feeding, rats were allowed to recover 180 min prior to being euthanized under CO_2_ gas. Of note, animals were injected (i.p.) with puromycin dihydrochloride 30 min prior to euthanasia (5.44 mg in 1 ml of diluted in phosphate buffered saline; Ameresco, Solon, OH, USA) to determine skeletal MPS levels via the SUnSET method [17].

Immediately following euthanasia, approximately two 50 mg pieces of mixed gastrocnemius muscle were harvested using standard dissection techniques and placed in RIPA buffer [Tris base; pH 8.0, NaCl, NP-40, sodium deoxycholate, SDS with added 1x protease (Ameresco) and 1x phosphatase II and III inhibitors (G BioSciences, St. Louis, MO, USA)] and Ribozol (Ameresco) for immunoblotting and mRNA analyses, respectively. Gastrocnemius samples placed in radioimmunoprecipitation assay (RIPA) buffer were homogenized immediately upon extraction using a micropestle, insoluble proteins were removed with centrifugation at 500 xg for 5 min, and supernatants were assayed for total protein content using a BCA Protein Assay Kit (Thermo Scientific, Waltham, MA, USA) prior to immunoblotting sample preparation. Gastrocnemius samples placed in Ribozol were subjected to total RNA isolation according to manufacturer’s instructions, and RNA concentrations were measured with a NanoDrop Lite (Thermo Scientific) prior to cDNA synthesis for mRNA analyses. Extra gastrocnemius muscle not processed during dissections was flash-frozen in liquid nitrogen and stored at −80 °C for later potential analyses.

### Directed Akt-mTOR phosphoproteomics

The PathScan® Akt Signaling Antibody Array Kit (Chemiluminescent Readout; Cell Signaling, Danvers, MA, USA) containing glass slides spotted with antibodies was utilized to detect phosphorylated proteins predominantly belonging to the Akt-mTOR signaling network.

Briefly, gastrocnemius homogenates were diluted to 0.5 μg/μl using cell lysis buffer provided by the kit and assayed according to manufacturer’s instructions. Slides were developed using an enhanced chemiluminescent reagent provided by the kit at 1 min exposure times for all chips, and spot densitometry was performed through the use of a UVP Imager and associated densitometry software (UVP, LLC, Upland, CA, USA). The calculation of each phosphorylated target was as follows:

(Density value of the target – density of negative control) / between-sample normalizing factor (which was the summation of all density values on the Chip for a given sample).

It should be noted that this high throughput antibody chip array for muscle phosphorylation markers was used rather than single antibodies due to resource constraints. Moreover, others in the literature have used chemiluminescent-based phosphoarray chips for screening purposes as we have utilized them herein [18, 19]. Finally, prior publications from our laboratory using this method has shown that different doses of whey protein feedings in rats increases p-rps6, p-mTOR, and p-p70sk6 (Additional file 1: Figure S1 in Mobley et al. [16]), and these arrays are sensitive at detecting differences in phospho-signaling events that occur in rodents in response to cardiac ischemia-reperfusion [20].

### Western blotting

As mentioned prior, the SUnSET method was employed in order to examine if different dietary blends differentially affected MPS. Briefly, gastrocnemius RIPA homogenates were prepared for Western blotting using 4x Laemmli buffer at a concentration of 2 μg/μl. Thereafter, 20 μl of prepped samples were loaded onto pre-casted 12 % SDS-polyacrylamide gels (Bio-Rad, Hercules, CA, USA) and subjected to electrophoresis (60 min @ 200 V). Proteins were then transferred (120 min @ 200 mAmps) to polyvinylidene difluoride membranes, and membranes were blocked for 1 h at room temperature with 5 % nonfat milk powder in Tris-buffered saline + Tween-20 (TBST). For muscle samples, mouse anti-puromycin IgG (clone 12D10, 1:5,000; Millipore-Merck KGaA, Darmstadt, Germany) was incubated with membranes overnight at 4 °C in 5 % bovine serum albumin (BSA) in TBST, and the following day membranes were incubated with anti-mouse IgG secondary antibodies (1:2,000, Cell Signaling, Danvers, MA, USA) at room temperature for 1 h prior to membrane development described below. Thereafter, membranes were stripped of antibodies via commercial stripping buffer (Restore Western Blot Stripping Buffer, Thermo Scientific), membranes were then re-blocked as described above and incubated with rabbit anti-beta-actin (1:5,000; GeneTex, Inc., Irvine, CA, USA) as a normalizer protein overnight at 4 °C in 5 % BSA in TBST, and the following day membranes were incubated with anti-rabbit IgG secondary antibodies (1:2,000; Cell Signaling) at room temperature for 1 h prior to membrane development.

Membrane development was performed using an enhanced chemiluminescent reagent (Amersham, Pittsburgh, PA, USA), and band densitometry was performed through the use of a gel documentation system and associated densitometry software (UVP, Upland, CA, USA).

### Real-time RT-PCR

Gastrocnemius RNA was obtained using Ribozol (Ameresco, Solon, OH, USA) per the manufacturer’s recommendations. Total RNA concentrations as well as RNA purity (260/280 OD ratio > 1.8) were analyzed using a Nanodrop Lite spectrophotometer (Thermo Scientific, Waltham, MA, USA), and 1 μg of cDNA was synthesized using a commercial qScript™ cDNA SuperMix (Quanta Biosciences, Gaithersburg, MD, USA) per the manufacturer’s recommendations. Following cDNA synthesis the resultant cDNA was diluted to 5 ng/μl using RNase-free water. Real-time PCR was performed using SYBR-green-based methods with gene-specific primers designed using primer designer software (Primer3Plus, Cambridge, MA, USA). The forward and reverse primer sequences are as follows: [Myostatin (Mstn): forward primer 5′- ACGCTACCACGGAAACAATC −3′, reverse primer 5′- CCGTCTTTCATGGGTTTGAT −3′; p21Cip1: forward primer 5′- AGCAAAGTATGCCGTCGTCT −3′, reverse primer 5′- ACACGCTCCCAGACGTAGTT −3′; Atrogin-1: forward primer 5′-CTACGATGTTGCAGCCAAGA −3′, reverse primer 5′- GGCAGTCGAGAAGTCCAGTC −3′; Muscle RING finger protein-1(MuRF-1): forward primer 5′- AGTCGCAGTTTCGAAGCAAT-3′, reverse primer 5′- AACGACCTCCAGACATGGAC-3′; Glucose transporter-4 (Glut-4): forward primer 5′-GCTTCTGTTGCCCTTCTGTC −3′, reverse primer 5′- TGGACGCTCTCTTTCCAACT −3′; Peroxisome proliferator-activated receptor gamma coactivator 1-alpha (Pgc-1α): forward primer 5′- ATGTGTCGCCTTCTTGCTCT- 3′, reverse primer 5′- ATCTACTGCCTGGGGACCTT −3′; β2-microglobulin (B2M; housekeeping gene): forward primer 5′- CCCAAAGAGACAGTGGGTGT - 3′, reverse primer 5′- CCCTACTCCCCTCAGTTTCC -3’] and SYBR green chemistry (Quanta). Fold-change values from the water (fasting) treatment were performed using the Livak method (i.e., 2-ΔΔCT assuming 100 % primer binding efficiency), where 2-ΔCT = [housekeeping gene CT – gene of interest CT] and 2-ΔΔCT (or fold-change) = [2-ΔCT value/2-ΔCT average of water (fasting) treatment]. Primer efficiency curves for all genes were generated and efficiencies ranged between 90 % and 110 %.

### Cell culture

C_2_C_12_ myoblasts (ATCC; Manassas, Virginia) were plated at approximately 40 % confluence and grown for 24 h in 10 % FBS High Glucose DMEM with antibiotics (100 μg/ml streptomycin and 100 U/ml penicillin; Sigma). At 18 h prior to the experiment, the myoblasts were switched to serum free high glucose DMEM (no antibiotics) and were approximately 90 % confluent at the time of the experiment. As detailed previously, the myoblasts were then stimulated with 30 μM of soy-derived PA (S-PA, Mediator®, Chemi Nutra, Austin, TX, USA) or PBS as a control condition [12]. For measurements of mTOR signaling, myoblasts were collected at 30 min after stimulation. For measurements of protein synthesis, 1 μM puromycin was added to the media during the final 30 min of a 60 min stimulation period, as previously described [17]. In all cases, myoblasts were collected in an ice-cold lysis buffer consisting of (40 mM Tris, pH 7.5; 1 mM EDTA; 5 mM EGTA; 0.5 % Triton X-100; 25 mM β-glycerophosphate; 25 mM NaF; 1 mM Na_3_VO_4_; 10 μg/mL leupeptin; and 1 mM PMSF) and then centrifuged at 500 x g for 5 min. The resulting supernatants were then subjected to Western blot analyses for puromycin, total p70s6k and phosphorylated p70s6k (Thr389) as detailed previously [12].

### Statistics

All data are presented as means ± standard error. For *in vivo* data, statistics were performed between treatments using an ANOVA with protected LSD *post hoc* comparisons when applicable in order to avoid an inflated type I error rate. For *in vitro* data, treatment comparisons were made using independent samples t-tests. All statistics were performed using IBM SPSS version 22.0 and significance was determined at *p* < 0.05.

## Results

### PA and PA + WPC increase 3 h post-feeding phosphorylation of select mTOR pathway substrates

**Fig. 1:**
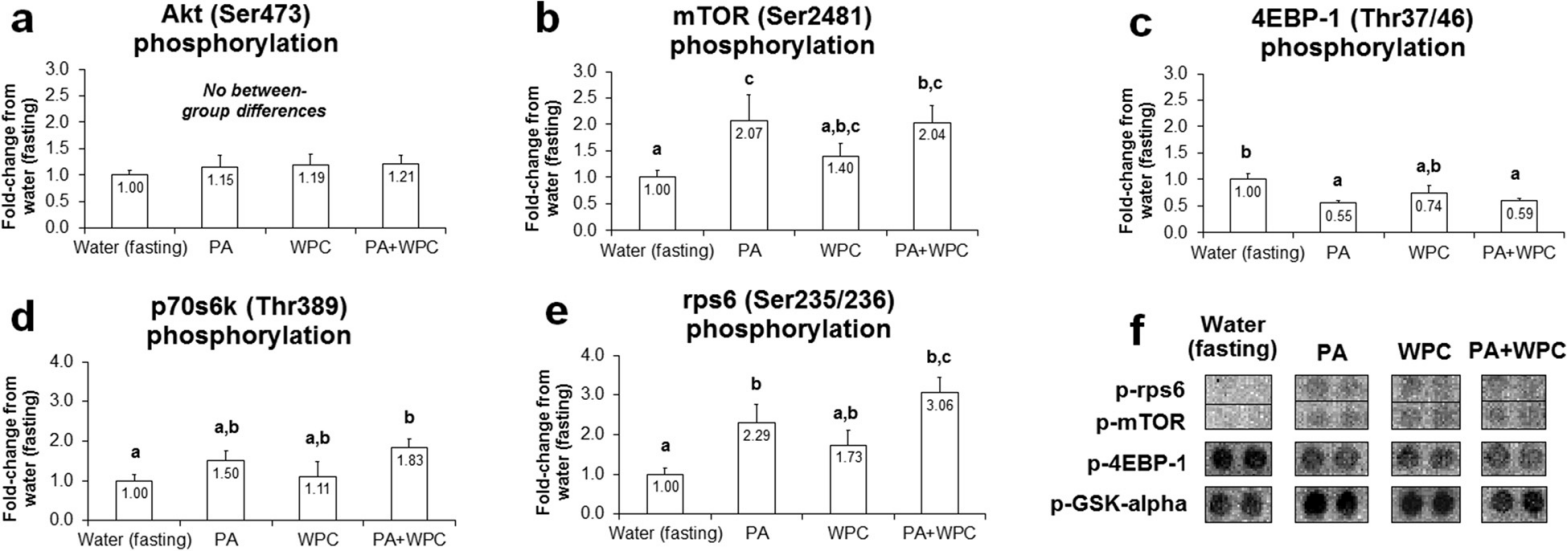
Effects of PA with or without the co-ingestion of WPC on mTOR signaling markers. Legend: Data are presented as means ± standard error, and bars not sharing similar superscript letters are significantly different (*p* < 0.05) as determined by one-way ANOVAs with protected LSD post hoc comparisons. CON n-size = 13, PA, WPC, and PA + WPC n-sizes = 7–8. Panels **a**–**e**: Effects of each ingredient on upstream positive modulators of protein synthesis. Panel **f**: representative digital images select phospho-targets that were altered with feedings

There was no difference in p-Akt (Ser473) between treatments (*p* > 0.05; Fig. 1a). Compared to CON, phosphorylated p-mTOR (Ser2481), which is a marker of mTOR autophosphorylation and activation [21], was approximately 2-fold higher in PA and PA + WPC rats (*p* < 0.05; Fig. 1b), though there was no difference in this marker between the latter two groups. Compared to CON, PA and PA + WPC led to a paradoxical decrease in p-4EBP1 (Thr37/46) (*p* < 0.05; Fig. 1c). Compared to CON, p-p70s6k (Thr389) was ~80 % higher in PA + WPC rats (*p* < 0.05; Fig. 1d), whereas other treatments did not statistically differ from CON. Compared to CON, p-rps6 (Ser235/236) was approximately 2.3–3.1-fold higher in PA and PA + WPC rats (*p* < 0.05; Fig. 1e), though again there was no difference in this marker between the latter two groups.

### PA and/or PA + WPC minimally affect 3 h post-feeding phosphorylation of other substrates related to protein synthesis

**Fig. 2:**
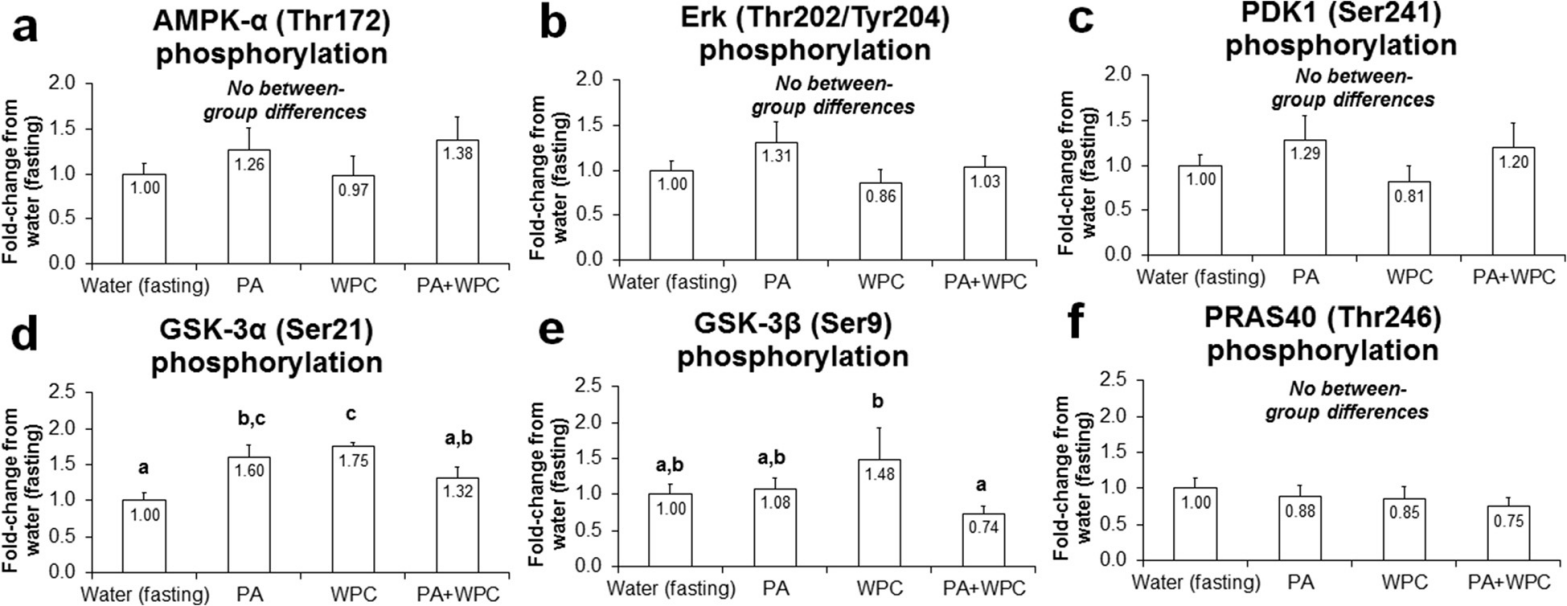
Effects of PA with or without the co-ingestion of WPC on other intramuscular signaling markers. Legend: Data are presented as means ± standard error. Bars not sharing similar superscript letters are significantly different (*p* < 0.05) as determined by one-way ANOVAs with protected LSD post hoc comparisons. CON n-size = 13, PA, WPC, and PA + WPC n-sizes = 7–8. Panel **a**: Effects of each ingredient on AMPK-α phosphorylation 3 h post-ingestion (cellular energy sensor; more phosphorylation is related to decreased mTOR pathway activation). Panel **b**: Effects of each ingredient on Erk-1/2 phosphorylation 3 h post-ingestion (positive upstream modulator of protein synthesis). Panel **c**: Effects of each ingredient on PDK1 phosphorylation 3 h post-ingestion (upstream modulator of protein synthesis). Panel **d & e**: Effects of each ingredient on GSK-3α/β phosphorylation 3 h post-ingestion (upstream modulator of protein synthesis). Panel **f**: Effects of each ingredient on PRAS40 phosphorylation 3 h post-ingestion (negative upstream modulator of protein synthesis)

All treatments equally affected the phosphorylation status of AMPK-α (Thr172), Erk (Thr202/Tyr204), PDK1 (Ser241), and PRAS40 (Thr246) (Fig. 2). Interestingly, PA increased p-GSK-3α (Ser21) 60 % compared to CON (*p* < 0.05; Fig. 2d). As reported previously [22], WPC increased the phosphorylation of GSK-3α/β (Fig. 2d & e), though PA + WPC interestingly exacerbated this response compared to WPC alone.

### Effects of PA and PA + WPC on 3 h post-feeding MPS levels

**Fig. 3:**
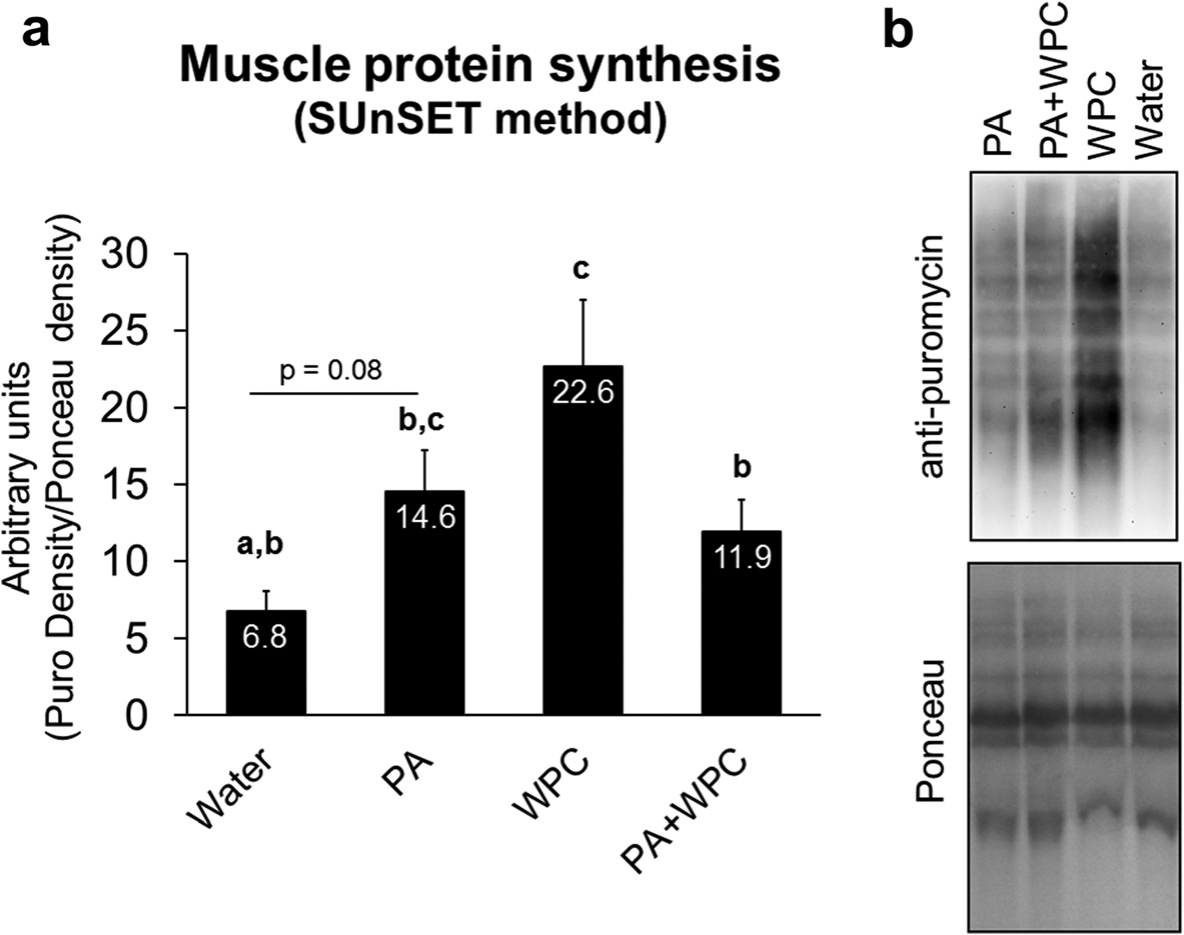
Effects of PA with or without the co-ingestion of WPC on 3 h post-feeding MPS. Legend: Data are presented as means ± standard error, and bars not sharing similar superscript letters are significantly different (*p* < 0.05) as determined by one-way ANOVAs with protected LSD post hoc comparisons. CON, PA, WPC, and PA + WPC n-sizes = 6–8. Panel **a**: Effects of each treatment on 3 h post-feeding MPS levels. Panel **b**: Representative image of the SUnSET blot. PA, WPC, and PA + WPC all significantly increased MPS levels compared to fasting conditions

Of note, WPC rats have been used in a prior study whereby we reported the significant two-fold increase in MPS relative to CON [22]. However, in the current study samples were re-run and normalized to Ponceau staining instead of beta-actin normalization given that mixed gastrocnemius fiber types may potentially yield varying normalization values. As expected, WPC robustly increased MPS levels compared to CON rats confirming our prior data. Compared to CON rats, MPS trended to be higher in PA-fed rats (*p* = 0.08; Fig. 3). Interestingly, however, PA + WPC-fed rats exhibited lower MPS levels compared to WPC-fed rats (*p* < 0.05, Fig. 3).

### In vitro confirmation that PA increases MPS and mTOR signaling

**Fig. 4:**
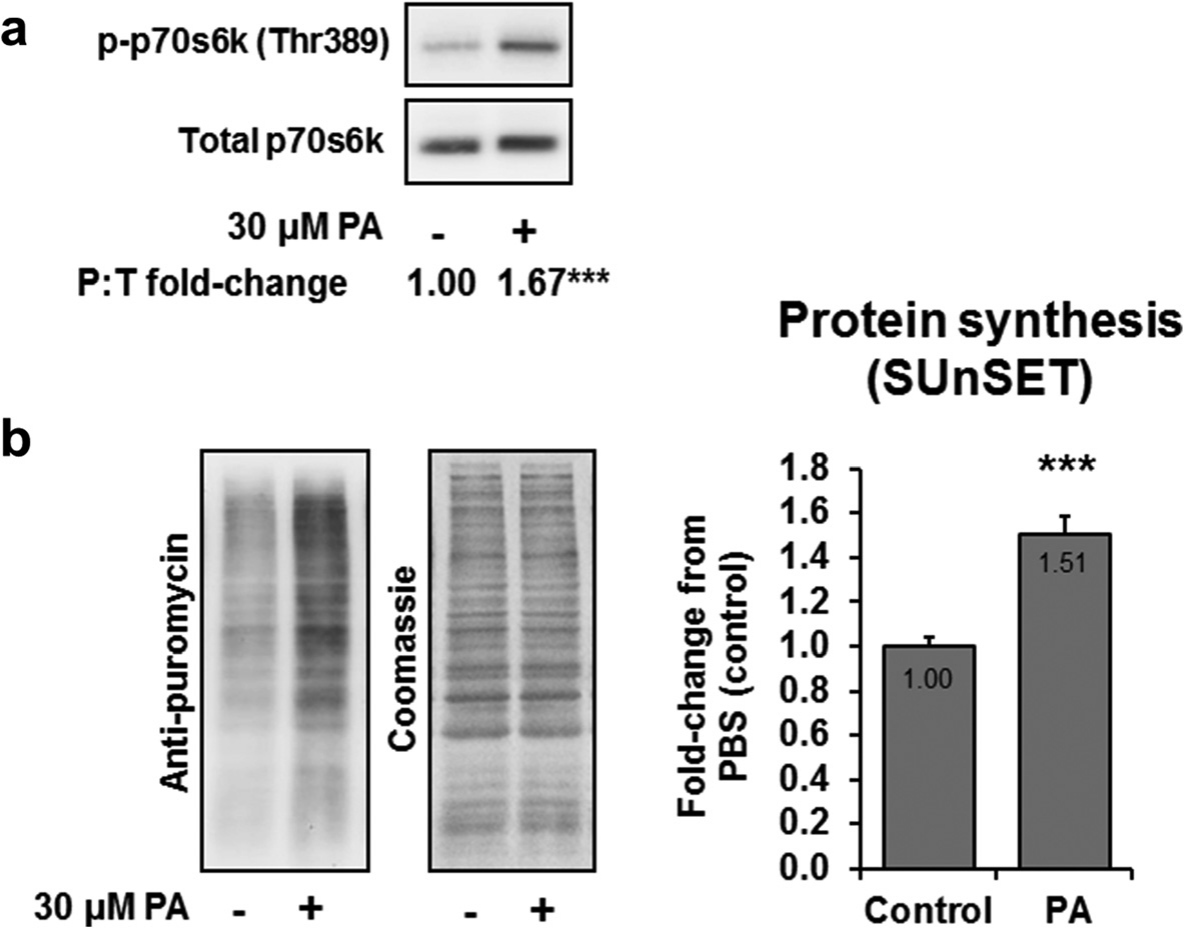
PA increases C_2_C_12_ myoblast mTOR signaling and MPS. Legend: Data are presented as means ± standard error. All data were obtained from 2–3 independent experiments (*n* = 6–11 wells per condition). Significant between-treatment differences (*p* < 0.001) existed for p-p70s6k (Thr389) and MPS levels as determined by independent samples t-tests (denoted by different superscript letters). Panel **a**: C_2_C_12_ myoblasts were serum starved for 18 h and then stimulated for 30 min with 30 μM PA, or PBS as a control condition. Measurements of mTOR signaling were assessed by evaluating the phospho to total ratio (P:T) for p-p70s6k (Thr389) and total p70S6k. Panel **b**: C_2_C_12_ myoblasts were treated as described in panel a and measurements of protein synthesis were performed by assessing puromycin incorporation during the final 30 min of a 60 min stimulation period. The Western blot membranes were stained with Coomassie Blue to verify equal loading of protein in all lanes

p-p70s6k (Thr389) increased 67 % in 30 min PA-treated myoblasts compared to the control condition (*p* < 0.001; Fig. 4a). Moreover, MPS levels were increased 51 % in 60 min PA-treated myoblasts compared to the control condition (*p* < 0.001; Fig. 4b). These data further validate the aforementioned *in vivo* results suggesting that the soy-derived PA studied herein increases mTOR signaling and likely increases MPS.

### PA and PA + WPC increase select 3 h post-feeding mRNAs related to skeletal muscle anabolism and metabolism

**Fig. 5:**
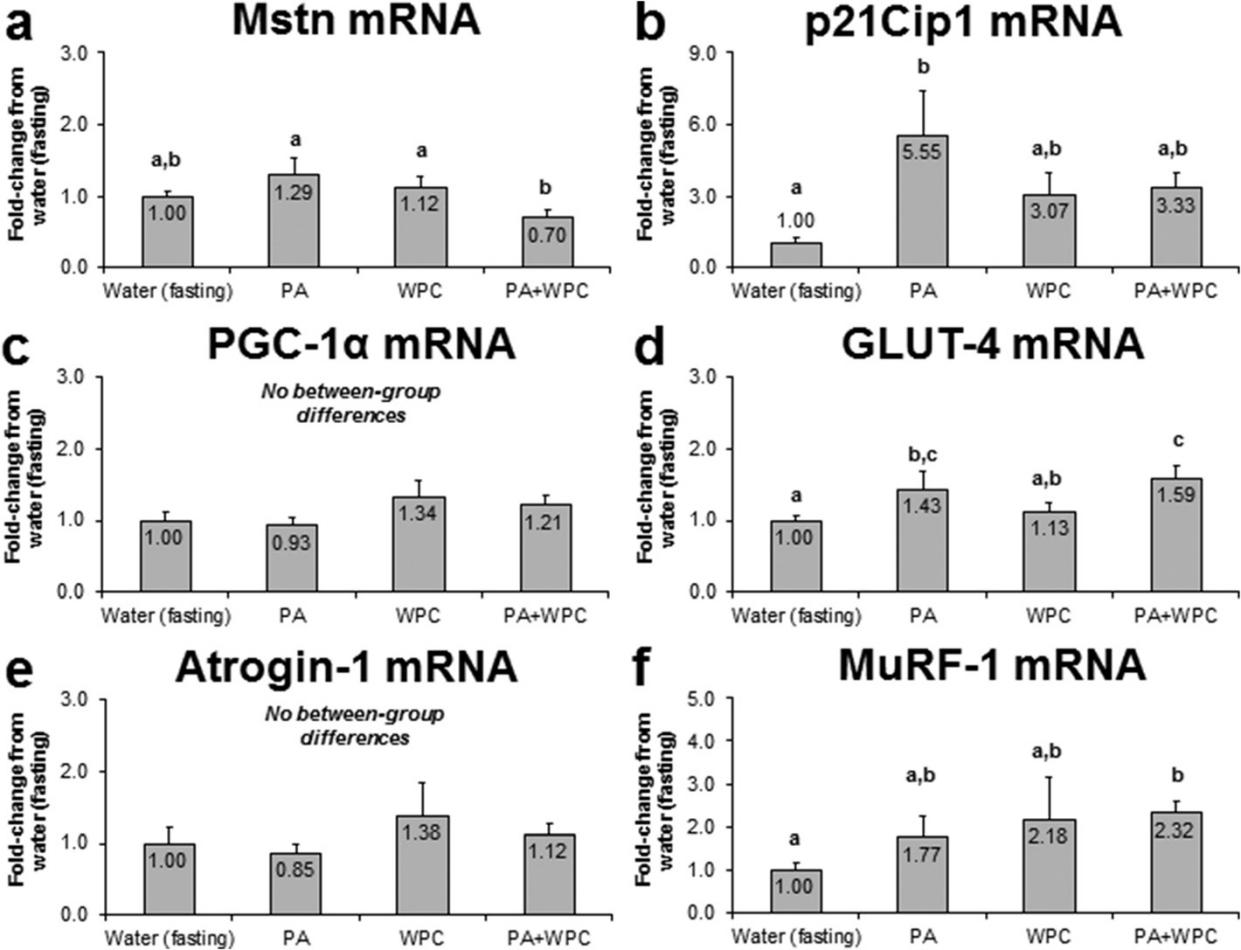
Effects of PA with or without the co-ingestion of WPC on skeletal muscle mRNA expression patterns. Legend: Data are presented as means ± standard error. Bars not sharing similar superscript letters are significantly different (*p* < 0.05) as determined by one-way ANOVAs with protected LSD post hoc comparisons. CON n-size = 13, PA, WPC, and PA + WPC n-sizes = 7–8. Panels **a & b**: Genes linked to skeletal muscle hypertrophy. Panels **c & d**: Genes linked to skeletal muscle metabolism. Panels **e & f**: Genes linked to skeletal muscle atrophy (Atrogin-1) and remodeling (MuRF-1)

Beyond examining the effects that PA with or without WPC exhibited on mTOR signaling and MPS, we also were interested in prospectively exploring if these treatments acutely affected gene expression patterns related to skeletal muscle mass regulation and metabolism. Interestingly, PA or WPC did not cause a change in Mstn mRNA compared to CON, though PA + WPC rats presented statistically lower Mstn mRNA levels relative to the PA- and WPC-fed rats (*p* < 0.05; Fig. 5a); however, Mstn mRNA in WPC + PA did not change compared to the control group. Compared to CON, PA robustly increased p21Cip1 mRNA expression patterns over 5.6-fold (*p* < 0.05, Fig. 5b). Likewise, compared to CON, WPC and PA + WPC increased p21Cip1 mRNA expression over 3-fold, though this only tended to be greater than fasting (*p* < 0.10) due to the high post-feeding variation in this transcript. No between-group differences existed in PGC-1α mRNA levels (Fig. 5c). Compared to CON, PA increased Glut-4 mRNA levels 43 % (*p* < 0.05; Fig. 5d) and PA + WPC increased Glut-4 mRNA expression patterns ~60 % (*p* < 0.05, Fig. 5d). All treatments presented similar Atrogin-1 mRNA levels (Fig. 5e). Finally, PA + WPC increased MuRF-1 mRNA 2.3-fold relative to CON (*p* < 0.05, Fig. 5f).

## Discussion

This is the first study to demonstrate that PA, with or without WPC, increases the post-feeding phosphorylation of select skeletal muscle mTOR pathway substrates, and PA tends to increase MPS levels *in vivo*. Moreover, our *in vitro* data demonstrate that exogenous PA administration increases mTOR signaling and MPS in C_2_C_12_ myoblasts. These data complement the two human studies suggesting that 8 weeks of PA supplementation increases skeletal muscle mass [11, 12]. These findings are discussed in greater detail below.

### PA, with or without WPC, increases post-feeding mTOR pathway activity in vivo

Our findings that PA, with or without WPC co-ingestion, increases post-feeding mTORC1 pathway activity and MPS *in vivo* supports other mechanistic literature examining the effects of PA on these variables. For instance, Fang et al. [5] treated HEK293 cells with PA and the authors demonstrated that: a) p70s6k activity increased; b) rapamycin (an mTOR inhibitor) blunted PA-induced increases in p70s6k activity; c) blunting serum-induced PA production in HEK293 cells with 1-butanol reduced p70s6k activity; and d) PA has a high binding affinity for mTOR on the FRB rapamycin-sensitive domain which, upon binding, increases the phosphorylation of mTOR-dependent substrates such as p70s6k (Thr389). The aforementioned study also demonstrated that PA levels in HEK293 cells had no effect on p-Erk1/2 (Thr202/Tyr204) or p-Akt (Ser473) levels.

The effects of PA in rat skeletal muscle presented herein are in agreement with the aforementioned data. Specifically, we demonstrated that: a) PA or PA + WPC did not alter 3 h post-feeding p-Akt (Ser473) or p-Erk1/2 (Thr202/Tyr204) levels relative to CON; and b) PA and PA + WPC increased p-mTOR (Ser2481) levels, as well as p-rps6 (Ser235/236) relative to CON. Furthermore, our *in vivo* data demonstrates that PA tended to increase MPS and PA + WPC significantly increased MPS levels relative to CON. However, it is interesting that, while WPC increased MPS the greatest, PA + WPC did not synergistically increase MPS levels in spite of the fact that p-p70s6k (Thr389) and p-rps6 (Ser235/236) were numerically the greatest in the combined fed group relative to PA and WPC alone. In this regard, we speculate that one of two phenomena are occurring: a) combined PA and WPC may alter the mTOR pathway activation dynamics relative to WPC-induced mTOR activation and, thus, peak MPS levels are shifted to the right or left of the 3 h post-feeding sampling point taken in the current study; or b) PA may actually interfere with WPC-induced increases in MPS. With regards to the latter point, PA and PA + WPC rats presented decreased levels of p-4EBP-1 relative to CON; this being a potential mechanism whereby PA may have ‘interfered’ with WPC-induced increases in MPS. This finding is difficult to reconcile as *in vitro* investigations have demonstrated that PA treatment [14] or inhibition of PA production [5] results in the increased and decreased phosphorylation of 4EBP-1, respectively. Thus, it will be of future interest to examine how PA and PA + WPC affect mTORC1 signaling, namely 4EBP-1 phosphorylation, and MPS levels over multiple post-feeding time points *in vivo*.

### PA, with or without WPC, differentially affect post-feeding skeletal muscle mRNAs related to anabolism and metabolism

Of the mRNA alterations that occurred with PA and/or PA + WPC, our major findings include: a) PA led to a robust post-feeding increase in p21Cip1 mRNA expression relative to CON; b) PA and PA + WPC led to a significant increase in Glut-4 mRNA expression relative to CON; and c) PA + WPC led to a significant increase in MuRF-1 mRNA expression relative to CON.

p21Cip1 gene expression is thought to promote satellite cell differentiation [23, 24], though others have shown that p21Cip1 gene expression is linked to protein synthesis and hypertrophy in post-mitotic kidney epithelial cells [25]. Furthermore, we [26] and others [27] have shown a 1–6 h robust increase in p21Cip1 mRNA expression after an acute bout of resistance exercise which suggests that this gene is being expressed from post-mitotic muscle fibers rather than differentiating satellite cells. Hence, it remains plausible that mTORC1 pathway activation may be linked to the mRNA expression of p21Cip1 which, in turn, may be related to enhanced translational mechanisms; this being a hypothesis which we have posited previously [28]. In support of this hypothesis, Nader et al. [29] have shown that mTOR increases the expression of cell cycle-related genes in post-mitotic myotubes in order to enhance ribosomal biogenesis. Therefore, it will be of further interest to examine if chronic PA supplementation leads to an increase in intramuscular markers of ribosome biogenesis, whether this effect is mitigated through an enhanced p21Cip1 gene expression, and whether this potential effect is causally-related to muscle hypertrophy evident with longer-term supplementation.

Our finding that PA + WPC ingestion increased MuRF-1 mRNA expression relative to CON is intriguing. While amino acids are anti-catabolic [30], other literature has shown that protein ingestion increases the mRNA expression of MuRF-1 mRNA in human skeletal muscle after resistance exercise [31, 32]. Conversely, skeletal muscle MuRF-1 mRNA as well as markers of muscle protein synthesis has been reported to be lower in spinal-cord-injured patients [33]; a finding which the authors suggest is linked to the tight regulation of muscle protein breakdown and synthesis rates. Thus, the results in the current study suggesting that PA + WPC ingestion increases the mRNA expression of MuRF-1 may represent a stimulation of greater muscle protein turnover (i.e., increased synthesis paralleled with increased breakdown rates) rather than an increase in atrophic mechanisms.

Finally, our finding that PA and PA + WPC modestly increased post-feeding Glut-4 mRNA is intriguing given the potential that this may be a manner whereby PA supplementation could potentially increase glucose sensitivity in an mTOR-dependent manner. To this end, insulin-like growth factor-1, a known activator of the mTORC1 signaling cascade, has been shown to increase Glut-4 mRNA *in vitro* [34]. Recent evidence also suggests that six weeks of cycling increases type II muscle fiber Glut-4 protein levels which were co-incident with increases in p-mTOR levels [35]. Thus, there seems to be interplay between mTORC1 activation and Glut-4 gene expression. To this end, it will be of further interest to examine if PA supplementation is capable of affecting serum glucose levels in rodent models or human populations with impaired glucose tolerance.

## Conclusions

In summary, this is the first study to demonstrate that PA, with or without WPC co-ingestion, increases mTORC1 pathway activation. Furthermore, these data demonstrate that PA ingestion tends to increase MPS levels *in vivo* relative to the fasted condition. While these findings are novel, this study is not without its limitations including: a) the limited post-feeding time point interrogation, b) the lack of intramuscular PA data, and c) the absence of other PA and/or WPC dosages as well as the lack of an exercise stimulus. With regards to the limitation ‘b’, we attempted to use a commercial fluorometric assay to measure muscle PA levels, but we did not have confidence in the data due to the high background fluorometric signal that the assay buffer possessed. With regard to the last limitation, future studies are needed in order to examine if: a) pre-loading with PA and/or higher doses of PA further promotes skeletal muscle anabolism, and b) if higher doses of PA in an acute exercise setting facilitates greater increases in MPS relative to the dose used herein. To this end, mechanistic rat studies in our lab using a hind limb stimulation model of resistance exercise has proven to be effective for increasing MPS in the fasted state (*unpublished observations*) and, thus, this model could be a valuable tool that continues to explore the efficacy of PA in enhancing post-exercise skeletal muscle anabolism. Given that mTORC1 pathway activation is integral in myoblast proliferation [36] and differentiation [37], examining how PA supplementation with resistance training affects myonuclear domain size, myonuclear number and satellite cell number is also warranted. It should also be noted that serum was not obtained from these animals and, given that Glut-4 mRNA transiently increases in PA-fed rats, future research should examine how dietary PA effects the post-prandial hormonal milieu (i.e., potential PA-mediated increases in insulin). Finally, the potential metabolic effects of PA supplementation beyond skeletal muscle hypertrophy (i.e., the effects of PA supplementation on glucose tolerance and/or appetite given that hypothalamic mTORC1 activation is anorectic [38]) may unveil PA as a nutraceutical candidate in populations beyond resistance-trained athletes.

## Abbreviations

4EBP-1/2: Eukaryotic initiation factor 4E-binding proteins 1/2
B2M: β2-microglobulin
CON: Control
FRB domain: FKBP12-rapamycin binding
Glut-4: Glucose transporter-4
LBM: Lean body mass
MPS: Muscle protein synthesis
mRNA: messenger RNA
Mstn: Myostatin
mTOR: Mammalian target of rapamycin
MuRF-1: Muscle RING finger protein-1
p70s6k: p70s6 kinase
PA: Phosphatidic acid
Pgc-1α: Peroxisome proliferator-activated receptor gamma coactivator 1-alpha
rps6: ribosomal protein s6
RIPA buffer: Radioimmunoprecipitation assay
WPC: Whey protein concentrate
